# Advances and Controversies in Acute Alcohol-Related Hepatitis: From Medical Therapy to Liver Transplantation

**DOI:** 10.3390/life13091802

**Published:** 2023-08-24

**Authors:** Giacomo Germani, Francesca D’Arcangelo, Marco Grasso, Patrizia Burra

**Affiliations:** 1 Multivisceral Transplant Unit, Azienda Ospedale—Università Padova, Department of Surgery, Oncology and Gastroenterology, University of Padova, 35122 Padova, Italy; 2 Gastroenterology and Multivisceral Transplant Unit, Azienda Ospedale—Università Padova, Department of Surgery, Oncology and Gastroenterology, University of Padova, 35122 Padova, Italy; francesca.darcangelo02@gmail.com (F.D.); marco.grasso.1994@hotmail.it (M.G.); burra@unipd.it (P.B.)

**Keywords:** alcohol, hepatitis, liver, transplantation, stigma

## Abstract

Alcohol-related hepatitis (AH) is a clinical syndrome characterized by recent-onset jaundice in the context of alcohol consumption. In patients with severe AH “unresponsive” to steroid therapy, mortality rates exceed 70% within six months. According to European and American guidelines, liver transplantation (LT) may be considered in highly selected patients who do not respond to medical therapy. The aim of this narrative review is to summarize current knowledge from medical therapy to liver transplantation in acute alcohol-related hepatitis. Due to the impossibility to guarantee six-month abstinence, LT for AH is controversial. Principal concerns are related to organ scarcity in the subset of stigma of “alcohol use disorder” (AUD) and the risk of relapse to alcohol use after LT. Return to alcohol use after LT is a complex issue that cannot be assessed as a yes/no variable with heterogeneous results among studies. In conclusion, present data indicate that well-selected patients have excellent outcomes, with survival rates of up to 100% at 24 and 36 months after LT. Behavioral therapy, ongoing psychological support, and strong family support seem essential to improve long-term outcomes after LT and reduce the risk in relapse of alcohol use.

## 1. Introduction

Alcohol-related hepatitis (AH) represents a unique challenge in the field of liver transplantation (LT), with a more than 70% mortality rate within 6 months in patients with severe AH and who do not respond to steroids therapy [1]. 

A literature search was performed from the PubMed bibliographic database using the following keywords: acute: “acute” OR “acutely” OR “acutes”; alcoholic hepatitis: “hepatitis, alcoholic” OR “hepatitis” AND “alcoholic” OR “alcoholic” OR “alcoholic” AND “hepatitis”; liver transplantation: “liver transplantation” OR “liver” AND “transplantation” OR “liver transplantation”. Overall, 369 articles written in English and published from 1988 to 2023 were identified. Titles and abstracts of the papers were evaluated by two authors (FD and MG). Articles on alcohol-related liver diseases that did not report data on acute alcoholic hepatitis were excluded and original articles and clinical trials on AH were selected. 

Although alcohol-related liver disease (ALD) is one of the most frequent indications for LT both in Europe and the USA, accounting for almost 20% of liver transplant recipients, LT for AH remains a controversial indication [2,3]. In this narrative review, we summarize current knowledge and future perspectives about LT in acute alcohol-related hepatitis (AAH). 

## 2. Definition and Prevalence

**Figure 1:**
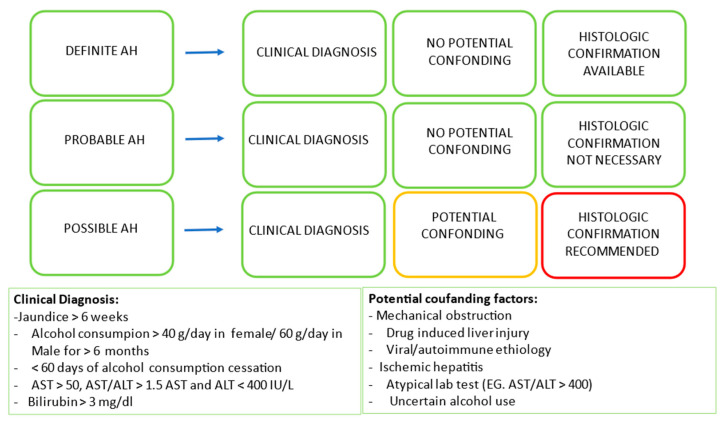
Diagnosis of alcohol hepatitis (AH). Definite AH: Clinically diagnosed and biopsy-proven. Probable AH: biopsy is not essential for diagnosis in the presence of clinical diagnosis in the absence of potential confounding. Possible AH: Clinical diagnosis with potential confounding factors and uncertain alcohol use, biopsy needed to confirm diagnosis.

AH has recently been defined by the National Institute on Alcohol Abuse and Alcoholism (NIAAA) Alcoholic Hepatitis Consortia as a clinical syndrome associated with newly onset jaundice (within 8 weeks) in the context of alcohol consumption in the amount of more than 40/60 gr of alcohol daily intake in female and male patients, respectively, in at least 6 months with less than 60 days of abstinence [4]. Frequently associated laboratory marker alterations are bilirubin >3 mg/dL and AST (>50 IU/mL)/ALT > 1.5, both with values < 400 IU/mL [4]. Potential co-founding factors such as mechanical obstruction and other causes of liver disease (i.e., drug-induced liver injury, viral hepatitis, autoimmune hepatitis, ischemic hepatitis) must be excluded. Based on imaging technique and blood test results, patients should be classified into definite, probable, and possible AH (Figure 1). The exact prevalence of AH is not acknowledged, but it is estimated to be increased, especially in young and female patients [5,6]. 

## 3. Pathogenesis and Predisposing Factors

**Figure 2:**
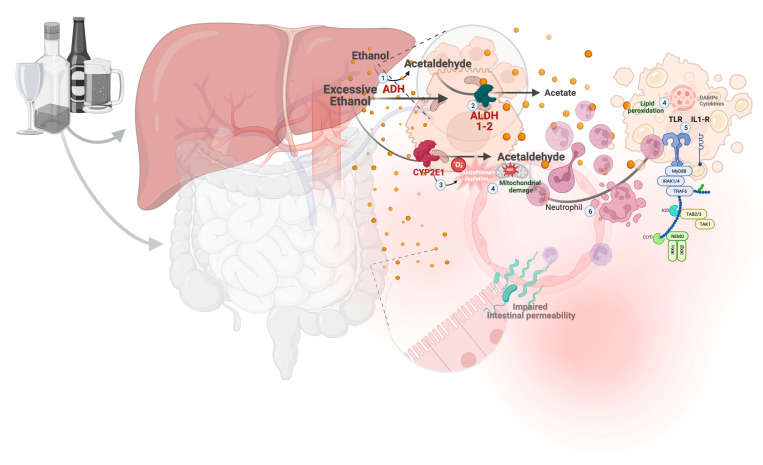
Pathogenesis of alcoholic hepatitis (AH). Shows the metabolism and mechanisms involved in hepatocyte dysfunction, including neutrophil cell-mediated damage and the role of the gut–liver axis. ADH denotes alcohol dehydrogenase; ROS, reactive oxygen species; CYP2E1, inducible enzyme. Created with Biorender.com.

Alcohol is mainly metabolized to acetaldehyde by alcohol dehydrogenase (ADH) (Figure 2, point 1) in the liver. Acetaldehyde is subsequently converted to acetate (non-toxic) by cytoplasmic aldehyde dehydrogenase one (ALDH1) and mitochondrial ALDH2 (Figure 2, point 2). In case of excessive ethanol intake, ADH activity is not sufficient; therefore, the inducible enzyme CYP2E1 (Figure 2, point 3) is activated and ethanol is transformed to acetaldehyde via the formation of reactive oxygen species (ROS) [7]. The metabolic products of ethanol are responsible of glutathione depletion, mitochondrial damage, and lipid peroxidation, resulting in hepatocyte injuries and production of damage-associated molecular patterns (DAMPs), cytokines, and inflammatory chemokines (Figure 2, point 4) [8]. These mediators binged to Toll-like receptors (TLRs) induce NLRP3 inflammasome activation and caspase 1 cleavage with interleukin (IL) -1β formation (Figure 2, point 5) [9]. The consequent hyperactivation of innate immune responses leads to neutrophil infiltrate (Figure 2, point 6) accumulation in the liver, known as the hallmark histological sign of AH. As recently highlighted, high quantities of ethanol have been reported to cause enterocyte junction destruction with increased intestinal permeability. This mechanism, in addition to alteration of microbial flora, may promote the translocation of microbial endotoxins (PAMPs), leading to the perpetuation of liver damage (Figure 2) [10]. Lastly, uncontrolled activation of the immune system leads to “immune exhaustion” that predisposes patients to early infections, systemic inflammatory response syndrome (SIRS), and onset of multiorgan failure (MOF).

Despite the increased rates of alcohol consumption reported worldwide, only a minority of heavy drinkers develop AH. In addition, the female population was more susceptible to develop alcohol-related diseases with a lower quantity of alcohol intake than men [11,12]. In fact, ADH baseline activity is known to be lower in women than in men, resulting in higher alcohol-related liver injury [13]. Furthermore, gonadal hormones during the menstrual cycle and exposure to birth control pills could be also play a role in different levels of ethanol between men and women [14]. The discrepancy between exposure to ethanol and clinical manifestation is related to individual susceptibility, environmental, genetic, and epigenetic factors [15]. In fact, gene polymorphisms such as the CYP2E1 gene appear to be involved in lowering the risk of alcohol-related liver damage and patatin-like phospholipase domain-containing protein 3 (PNPLA3) in an increased risk of severe AH [16,17,18]. Moreover, the PNPLA3 gene polymorphism seems to be involved in the development of chronic non-alcohol-related liver damage and may be responsible for the development of long-term liver complications in patients who have experienced episodes of AH [16,17,18].

### The Link between ALD and MASLD

Alcohol-associated liver disease (ALD) represents a spectrum of liver lesions resulting from alcohol use, ranging from liver steatosis (SLD) to more advanced forms such as alcoholic hepatitis (AH), alcohol-associated cirrhosis (AC), and acute AH presented as chronic liver failure. A multi-society Delphi consensus statement recently updated the nomenclature regarding the spectrum of diseases associated with SLD [19]. Metabolic dysfunction-associated liver steatosis (MASLD) was created to include patients with liver steatosis and at least one of the five cardiometabolic risk factors, and a new category outside of pure MASLD was named MetALD [19]. MetALD includes individuals with MASLD who consume significant amounts of alcohol per week (140 g/week and 210 g/week for women and men, respectively) [19]. These conditions have similar pathological spectra, ranging from liver steatosis to steatohepatitis, liver cirrhosis, and hepatocellular carcinoma; differences in disease progression may be caused by various genetic and environmental factors that differ between individuals. The rs738409 G polymorphism of PNPLA3, which is involved in the coding of a protein involved in the hydrolysis of triacylglycerols, is one of the few genetic factors that increase patients’ vulnerability to the development and progression of both ALD and MASLD diseases [20,21,22]. Recent evidence shows that patients with MetALD and pure ALD have higher risk of developing decompensated cirrhosis when compared with patients with MASLD disease [23]. 

## 4. Long-Term Liver Complications of AH

Patients with moderate–severe AH have a one-month mortality rate of 23%, whereas liver failure, gastrointestinal bleeding, and infections are the leading causes of death [24]. Patients with severe AH may develop portal hypertension in the early stage of the disease due to altered hepatic venous drainage resulting from massive liver inflammation, or later with the concomitant development of cirrhosis with severe AH having a 10–20% risk per year of developing cirrhosis [25]. Moreover, Alexander JF et al. reported 40% of histologically diagnosed cirrhosis in a cohort of patients with AH episodes resolved with medical therapy in five years follow-up time [26]. The likelihood of progression of ALD after a single episode of AH appears to be associated with sustained alcohol use in a dose-dependent manner, but other factors could be involved in the risk of disease progression. Pares et al. reported disease progression to cirrhosis in absence of alcohol use in 57% (4/7) of women but not in men (0/6) in a small court of 26 patients (14 males and 12 females). Moreover, an underlying ALD in patients with prior AH can significantly increase the risk of developing HCC, which is estimated to be 3.6 times higher in patients who continue to use alcohol use when compared to patients who abstain entirely from alcoholic beverages [27]. Furthermore, a single nucleotide polymorphism (SNP) in the DNA of PNPLA3 has been reported to increase susceptibility to the development of HCC in patients with ALD [28,29].

## 5. Severity Assessment and Prognostic Models

Several prognostic scores have been developed to identify patients with AH at higher risk of early death. The modified Maddrey score (mDF) seems capable of identifying patients with high short-term mortality, and its use is currently recommended by European and American guidelines. A mDF cut-off value of 32 is used to identify patients with severe AH who require medical therapy (steroid-based) [30,31]. Another score currently in use is the Glasgow alcoholic hepatitis score (GAHS) computed by five variables (age, hematic levels of bilirubin, urea, and white cell count), which has been validated in the United Kingdom and seems able to more accurately identify patients who may benefit from steroid therapy than the mDF score [32]. In the context of AH, the MELD score has only been examined by retrospective studies that indicated an elevated risk of death within 90 days in patients with MELD > 20 [33]. In a retrospective cohort of 202 patients with AH, MELD score variation in the first week of disease was an independent predictor of in-hospital mortality (OR 1.3 (95% CI: 1.1–1.4), *p* < 0.0001) [33]. Moreover, Morales Arraez investigated the accuracy of the different scores currently available to predict short term mortality in 3101 patients with suspected AIH from 11 centers. The MELD score was reported to the be significantly superior to mDF in predicting mortality at 28 and 90 days [34]. Recently, Arab highlighted that MELD between 25–39 is the optimal therapeutic window for corticosteroids use in AH, reporting a decreased mortality rate at 30 days [35]. Severe AH has been associated only with improved short-term mortality with steroid therapy and scarce results on long term survival [36]. Therefore, liver transplantation remains the best treatment choice in well-selected patients with severe AH who do not respond to medical therapy. In this context, early identification of “non-responders” is crucial to improve outcomes. The Lille model considers pre-treatment data as follows: age, creatinine, albumin, PT, initial value of bilirubin and its value after seven days of steroid therapy; a Lille score > 0.45 identifies patients as “non-responders” [1]. The 4-day Lille score was also created in order to early identify “non-responsive” patients, although further validation is still needed [37]. A multicenter study conducted on 538 patients with severe AH treated with steroids compared three models (Maddrey + Lille, MELD + Lille, and ABIC + Lille) to identify the best score combination associated with better prognostic value. The MELD + Lille combination was found to perform better in predicting survival compared to the other models [38].

## 6. Medical Management 

### 6.1. Abstinence

Alcohol abstinence is the main prognostic factor associated with improved long-term survival, regardless of severity, and European and American guidelines recommend it as the first treatment strategy for alcohol-related liver disease [31,36]. Moreover, in patients with AH occurring in a non-cirrhotic liver, abstinence could also prevent fibrosis development and progression.

### 6.2. Corticosteroid Therapy

AH is an immune-mediated disease, with tumor necrosis factor (TNF) being the central mediator of damage progression. Glucocorticoids are currently the standard of care in AH due to their anti-inflammatory mechanism of action; however, the data are controversial and steroid therapy appears to only partially improve short-term mortality. In 1996, Mathurin et al. first reported that corticosteroids improved 1-year but not 2-year survival in a cohort of patients with AH treated for one month. Furthermore, hepatic polymorphonuclear infiltrates and polymorphonuclear count were reported to be independent prognostic factors, proving the role of inflammation in damage progression [39]. Conversely, Imperiale et al. conducted a meta-analysis of 11 RCTs (10 vs. placebo), reporting that steroid therapy was associated with decreased short-term mortality only in AH patients with hepatic encephalopathy and who never experienced digestive bleeding episodes [40]. Subsequent meta-analyses were not able to demonstrate significant survival benefits after corticosteroid therapy in patients with AH. However, the high heterogeneity of the study populations must be considered as a potential cause of these results [41,42,43]. In the systematic review by Gluud et al. (15 trials, 721 patients), the beneficial effect of steroids was observed only in patients with a Maddrey score > 32 or hepatic encephalopathy [43]. Lastly, the largest randomized trial to date (STOPAH) reported a decrease in 28-day mortality in patients with severe AH (treated with 40 mg prednisolone compared with a placebo; however, this improvement was not seen at 90 days and at one year [44]. The current European guidelines suggest the initiation of steroid therapy (prednisolone 40 mg/day or methylprednisolone 32 mg/day) in patients with severe AH (mDF ≥ 32 or GAHS ≥ 9) to reduce short-term mortality, emphasizing their ineffectiveness on mid- and long-term survival [36].

### 6.3. Nutritional Therapy

During severe AH, systemic inflammation and oxidative stress from excessive use of ethanol promote the development of malnutrition. In the study by Mendenhall et al., protein energy malnutrition in patients with severe AH was associated with a significant increase in 6-month mortality (*p* = 0.0012) [45]. Furthermore, in a randomized trial of 136 patients with severe AH, it was reported that patients with a daily intake of less than 21.5 kcal/kg/day had higher mortality than patients with a higher caloric intake (65% vs. 33.1%, *p* < 0.001) [46]. Adequate nutritional intake should be considered among the goals of medical treatment in patients with severe AH. The European Association for the Study of the Liver (EASL) recommends a daily protein intake of 1.2–1.5 g/kg and 30–40 kcal/kg body weight of energy in patients with AH. Although no differences in survival have been observed between different modes of feeding, European guidelines strongly recommend the use of a nasogastric tube in patients who cannot feed orally [46]. In patients with severe AH and concomitant signs of progression to liver failure with the onset of complications such as hepatic encephalopathy (HE), the use of branched-chain amino acids (BCAAs), in combination with adequate nutrition, as discussed above, has been shown to improve quality of life, increase survival, and reduce the progression of liver injury [47,48,49,50]. It is currently acknowledged that adequate protein support can be administered safely in HE patients to improve nitrogen balance and prevent the development of sarcopenia, which is associated with worsening post-liver transplant survival results and an increased risk of complications such as infections [49,51].

### 6.4. Emerging Therapies for AH

Targeted therapies have been investigated to supply the limited efficacy of steroid therapy in treating severe AH. Anti-TNFα drugs have been first explored in considering whether the role of TNF-mediated inflammatory drugs is responsible for damage progression. However, higher mortality rates associated with the increased risk of infection development have been reported [52,53]. Another attempt was made with Pentoxifylline, which reduces intracellular cyclic AMP levels and, consequently, inflammatory cytokines. Although initial studies reported the potential efficacy of this molecule, the most extensive study to date did not demonstrate any benefit of pentoxifylline on mortality over placebo, even when administered with steroid therapy [44]. Considering that inflammasome activation and IL-1 generation improve the progression of inflammation and damage in AH, the role of the primary IL-1 inhibitor (Anakinra) in AH was studied. However, no survival advantages were reported compared to steroid therapy [54]. Some studies have reported the potential effect of N-acetylcysteine when combined with steroids to improve survival [55,56]. 

Improved survival at 3 and 6 months was reported when steroids were combined with Methadoxin by Higuera et al. Methadoxin is an antioxidant capable of restoring glutathione stores in liver cells [57]. Moreover, a potential role in alcohol dependence therapy has been reported with a significant reduction in alcoholic drinks per week (*p* < 0.001) and lower cravings (assessed by visual analogue scale VAS) (*p* < 0.001) [57]. Finally, multiple clinical trials are currently (NCT03157388, NCT02281929, NCT02326103) investigating the role of the gut–liver axis on the pathogenesis of AH. Promising results are expected from microbiota transplantation, which could reduce the alteration of intestinal permeability and the progression of inflammation that occurs in AH. Moreover, a pilot study by Sarin et al. reported how fecal microbiota transplantation (FMT) from healthy donors was effective and safe in patients with severe AH, resulting in improved 1-year survival compared with controls (87.5% vs. 33.3%; *p* = 0.018) [58]. Emerging therapies for AH are summarized in Table 1.

## 7. Role of Liver Transplantation

Liver transplantation represents the best therapeutic option for patients with severe acute alcohol-related hepatitis (sAH) who do not respond to medical therapy [1]. Although European and American guidelines have recently stated that LT may be considered in highly selected patients who do not respond to medical therapy, policies for early liver transplantation (eLT) in patients with sAH are different among countries [31,36,59]. The Spanish Society for Liver Transplantation has recently included acute alcohol-related hepatitis as the first event of liver decompensation as a new potential indication for LT with a particular recommendation on psychosocial assessment [60]. Conversely, in Germany, pre-LT 6 months abstinence is mandatory and exceptions need to be approved by a specialist committee [61]; in Canada, LT for sAH is not performed [62]. In the USA, the number of centers performing LT for AAH is currently increasing from 14 in 2014 to 47; in France, 88% of liver transplant centers are reported to perform LT for AAH in 2018 [63,64].

## 8. Outcome after Early Liver Transplantation

### 8.1. Survival

The first data about LT in severe AH was reported by Mathurin et al. in 2011 in a multi-center Franco-Belgian pilot study, which included 26 patients with biopsy-proven AH and no response to steroid therapy [65]. The authors performed a strict selection criterion oriented on the patient’s social background, as reported in Table 2. Patients who underwent eLT presented significantly higher 6 months (77 ± 8% vs. 23 ± 8% *p* < 0.001) and 2 years survival rates (77 ± 8% vs. 23 ± 8% *p* < 0.001) when compared to matched control patients with sAH not responding to steroids therapy and who were candidate to LT [65]. No differences in terms of survival were observed comparing these 26 patients with matched controls who responded to medical therapy (77 ± 8% vs. 85 ± 4%, *p* = 0.33). After the results reported by Mathurin et al., others single-center studies were performed both in the USA and Europe [66,67,68,69,70,71]. The subsequent prospective study was performed by Im et al. in a cohort of 9 American patients with severe AH (mostly not biopsy proven) in 2016. Patients who underwent early LT were reported to have a 6 month survival rate that that was significantly higher when compared to matched controls excluded from early LT (89% vs. 11%, *p* < 0.001), with no occurring death in a median follow-up of 735 days (range, 181–1170 days) [66]. Later, Lee retrospectively compared 17 patients who underwent LT after a diagnosis of severe AAH with 26 patients affected by alcohol-related liver cirrhosis after 6 months of abstinence from a single American center, observing no difference in 6 months survival (100% vs. 88% *p* = 0.27) [67]. Despite the clinical diagnosis, explant histology revealed liver cirrhosis in 15 out of 17 patients (88%) [67]. Similar data were also observed by Lee et al. in a retrospective study of 147 consecutive patients with clinical diagnosis of severe AH from 12 different US centers, in which 40% of explant livers were found to have cirrhosis characteristics [68]. Furthermore, also in the Italian cohort reported by Germani et al. of 16 patients with clinically diagnosed AAH, explant histology revealed a pattern suitable for AH on subset of cirrhosis in all patients [71]. These findings may suggest that pure AAH is rare and occurs in pre-existing liver disease [72]. With respect to the long-term survival rate, satisfactory results have been reported. In the American series, Lee et al. first observed a 3 years survival rate of 84% (95% CI, 75–90%) in patients with early LT for AAH [68]. Data confirmed in 2022 where 95%, 88%, and 82% of 153 early transplanted patients, from 11 American centers, who were reported to be alive at 1, 3, and 5 years of follow up, respectively [69]. In the European series, Louvet et al. reported that 90% of patients who underwent early LT were still alive 2 year after LT, resulting in a four times higher improvement in survival compared with patients who were denied LT [70]. Finally, in Italy, Germani et al., in the second largest European experience, observed a 100% higher survival rate in the early LT cohort compared with responders to medical therapy and patients not suitable for LT 24 and 36 months after LT (100% vs. 38% and 35%, respectively; *p* < 0.001) [71]. 

### 8.2. Return to Alcohol Use after Liver Transplantation

Return to alcohol use after LT represents the main concerns for patients with alcohol-related liver disease, especially in the setting of AH where 6 months alcohol withdrawal cannot be guaranteed. Historically, in patients with alcohol-related liver disease, a period of 6 months of abstinence was required to select patients for admission to LT waiting lists [73]. However, this criterion has been questioned due to the lack of a solid scientific background [74,75]. As reported by Di Martini et al. in their prospective study, while longer alcohol abstinence before LT was found to be a predictor of lower alcohol relapse after LT, there is no defined time threshold that can ensure sobriety [76]. In the study by Mathurin et al., no patients resumed alcohol consumption within 6 months after LT, although three patients (11%) experienced alcohol relapse after 720 days of follow-up with no consequences in graft function [65]. In 2017, Lee BP reported no difference in return to alcohol use rate between early LT group and patients who underwent LT after 6 months of abstinence (23.5% vs. 29.2%, *p* = 0.99) [67]. Data from the American consortium of Early Liver Transplantation for Alcoholic Hepatitis (ACCELERATE-AH) showed a 10% and 17% rate of sustained alcohol use at 1 and 3 years from after LT, respectively [68]. Factors associated with decreased survival were the result of more than 10 drinks per day before LT presentation (HR, 3.17; 95% CI, 1.04–9.67; *p* = 0.04) and both any (HR, 3.54; 95% CI, 1.06–11.85; *p* = 0.04) and sustained (HR, 4.59; 95% CI, 1.45–14.54; *p* = 0.01) return to alcohol use after LT [68]. In 2019, the same group proposed the Sustained alcohol use post liver transplant-score (SALT-score) based on four variables, as reported in Table 3.

**Figure 3:**
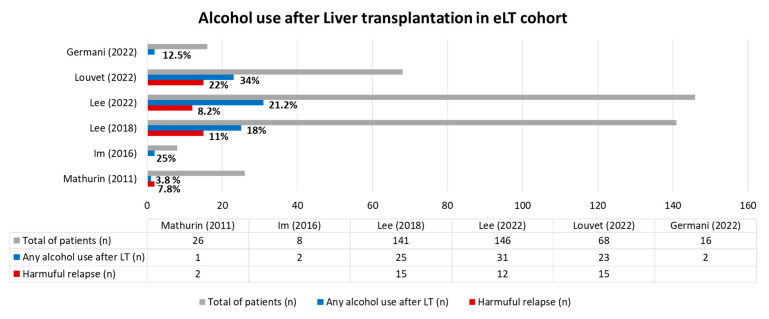
Alcohol use after liver transplantation in patients with AAH who underwent eLT. Louvet (2022) reported a pattern of alcohol use after LT during 2 years of follow up [70]. Lee (2018) reported alcohol use in a median follow up of 1.6 years [68]. Lee (2022) reported a different pattern of alcohol use after LT: early/heavy alcohol use (*n* = 12; 8.2%); in the table, any alcohol use (*n* = 31; 21.2%) referred to the sum of late/non-heavy pattern (*n* = 9) plus early/non-heavy (*n* = 22) [69]; in Im’s study (2016) of 8/9 patients survived after 6 months of follow up, 2 patients returned to alcohol consumption, of which 1 has ongoing alcohol use at the end of follow up [66]. Mathurin (2011) reported 3 patients who resumed alcohol use, of which 2/3 were daily consumers after 720 days of follow up [65]. Germani did not specify the pattern of alcohol use after LT [71].

However, this score, seems to only properly identify patients with a low risk of sustained alcohol use after LT, especially in those with SALT score < 5, who have a 95% negative predictive value for sustained alcohol use post-LT [77]. Similar findings were reported by Weinberg, who explored factors associated with harmful alcohol relapse after LT by comparing patients who underwent LT after a first event of liver decompensation vs. patients with history of alcohol prior decompensation [78]. Prior liver disease decompensation, history of failed rehab, and past use of more than 10 drinks per day were associated with an increased risk of harmful alcohol use after LT [78]. Based on these findings, return to alcohol use after LT is a complex issue that cannot be assessed as a yes/no variable. Recently, return to alcohol use after early LT has been classified into four different patterns: abstinence, late/non-heavy, early/non-heavy, early/heavy [69]. No difference in 1 year survival rate was observed between the 4 groups, although patients with early/non-heavy and with early/heavy patterns experienced lower 3- and 5-year survival rates when compared to abstinent and late/non-heavy drinkers [69]. Pre-LT characteristics associated with harmful return to alcohol use were younger age, prior multiple attempts of rehabilitation, and presence of hepatic encephalopathy [69]. Another attempt to classify return to alcohol use after LT was made by Louvet et al. using the Alcohol Timeline Followback (TLFB) instrument, which measures time spent consuming any and harmful amounts of alcohol [70]. Louvet compared return to alcohol use in eLT vs. the standard LT group (34% vs. 24%, respectively) [70]. Finally, no difference was observed regarding time spent in any alcohol use between the two groups; however, eLT patients were found to spend more time consuming a high quantity of alcohol (standardized difference 0.50; 95% CI 0.17–0.82) [70]. Promising results are expected in the future using artificial intelligence to stratify the risk of alcohol relapse after liver transplantation [79]. Figure 3 summarizes data on alcohol relapse from different studies.

### 8.3. Impact on LT Activity and Ethical Concern

Patients with severe AH are frequently admitted to LT waiting lists with higher MELD scores, which guarantee them shorter time on the WL compared to other etiologies of liver disease, raising ethical issues regarding the current WL management [72]. Bittermann recently reported a 6.5 fold increase in waitlist admission for AAH in the United Network for Organ Sharing (UNOS) area in 2008–2019 with 10 days of median waiting times for LT [80]. Moreover, patients with AAH and MELD > 30 had higher transplant rates when compared with patients with same value of MELD but not alcohol-related etiology (adjusted sub hazard ratio, 0.67; 95% CI, 0.52–0.86; *p*  =  0.002) with consequent reduced waitlist mortality [80]. Furthermore, patients with AH were be younger (43 years of median age vs. 51 years for other indications) and had higher levels of education [80]. However, Cotter et al. recently observed how the increased rate of transplant activity for AH has occurred in a setting of geographical disparities between UNOS regions, probably due to the lack of standardized criteria for patients’ selection [63]. Similarly, Im reported that the referral activity from other hospitals has increased up to 13% between 2011 and 2016, mainly due to AAH, which rapidly became the first reason for transfer request [81]. Conversely, the actual number and proportions of LTs performed for AH in the United States seems to be underestimated. In fact, Lee et al. compared data from the UNOS diagnosis and ACCELERATE database, with only 35% of AAH diagnosis reported in the UNOS registry [82]. Finally, in consideration of the increased number of alcohol-related hospitalizations during the COVID19 pandemic, Bittermann compared rates of WL admission before the pandemic (March 2018–Febraury 2020) with WL admission during COVID-19 (March 2020-Febraury 2021) [83]. A 106.6% mean increase in waiting list admission for AAH and 210.2% in liver transplantation performed were observed during the COVID-19 pandemic, compared with the pre-pandemic era (*p* < 0.001) [83]. Although there were good outcome rates of LT for AAH and younger age, these particular LT population ethical issues are still under debate. The main argument for AAH as an indication for LT is derived from the lack of standardized criteria for WL admission and the possible negative impact on organ donation by public perception of wasted resources for self-convicted disease [84]. For this purpose, Stroh distributed a survey to 503 currently living people in the USA (67.8% with intention to be an organ donor and 21.1% unsure) regarding LT for patients with AAH. Only the minority of responders (26.3%) admitted that the acknowledgement of possible LT for AAH would made them change their intention regarding organ donation; furthermore, the majority (81%) were neutral towards this information. Good social support and younger age were the factors most influencing the decision to proceed to LT [85]. Patients with alcohol-related liver disease, especially in the context of acute complexity of alcohol-related hepatitis, are broadly exposed to public stigma, self-stigma, and structural stigma impacting healthcare resource access with consequent negative outcomes [86]. Stigma of alcohol disease is reported to be more severe, even compared with other mental diseases or medical conditions caused by smoking habits or obesity; moreover, healthcare workers who were reported to moralize on ALD patients regarding their self-inflicted disease were involved [87,88,89].

## 9. Discussion

Several prognostic scores have been identified to predict patients’ survival during AH episodes, with Maddrey score being most capable to identify patients at higher risk of death, even though the MELD score was recently reported to be superior in predicting mortality at 28 and 90 days [30,31,34]. Furthermore, MELD score between 25–39 seems to be the optimal therapeutic window for corticosteroids use [35]. Glucocorticoids are the standard of therapy in AH, although they are ineffective in medium- and long-term mortality [36]. Emerging therapy has been investigated, with pentoxifylline being recommended by the EASL guidelines in patients with ongoing sepsis in whom corticosteroids are not feasible, and N-acetylcysteine is considered useful in association with corticosteroids [36]. Promising results are expected from ongoing clinical trials investigating the gut–liver axis. In this setting, liver transplantation remains the best therapeutic option for patients with severe AH who do not respond to medical therapy. Although European and American guidelines acknowledge liver transplantation in high selected patients, there is atherogenicity between countries such as France and the United States that have experienced an increase in LT activity for sAH and countries with more restricted criteria such as Germany or Canada, who do not perform LT for sAH [31,36,59,61,62,63,64]. From the Mathurin pilot study, subsequent investigations both in Europe and in the USA revealed a significantly higher survival rate for patients who underwent early LT, from 6 months to 2 years, compared to matched controls who do not respond to steroid therapy excluded from early LT [65,66,67,68,69,70,71]. However, explant histology revealed that AH in a subset of cirrhosis is more frequent than pure AH [69,71,72]. Therefore, return to alcohol use remains the main concern in this population when 6 months of abstinence cannot be guaranteed. The 6-month rule has been challenged due to a lack of scientific background in its ability to define a threshold that can guarantee sobriety after LT [74,75,76]. Multiple factors influence the return to alcohol use after LT, as experienced in the cohort by Mathurin and Germani, who reported a low rate of alcohol return after LT (10% and 12.5%) by applying strict selection criteria such as the presence of supportive family members or the exclusion of psychiatric comorbidities [65,71]. Attempts to predict and stratify the risk of alcohol relapse, such as the Sustained Alcohol Use Post Liver Transplant Score (SALT score), have shown promising results, but still need to be tested in a prospective cohort of patients [33]. Moreover, Louvet et al. tried to quantify return to alcohol use using the Alcohol Timeline Followback (TLFB) instrument, observing no difference in time spent consuming alcohol between the standard LT vs. eLT cohorts of patients, although eLT patients were found to spend more time consuming high quantities of alcohol [70]. From this evidence, return to alcohol use represents a much more complex issue that is difficult to characterize as a dichotomous variable (yes/no).

## 10. Conclusions

In conclusion, this review offers a comprehensive analysis of the current literature on severe acute alcohol-related hepatitis considering clinical implications, survival outcomes, and ethical concerns. However, some limitations need to be acknowledged. First, it is not conducted in a systematic way; second, we cannot conduct any pooled analyses using the data from the summarized studies. Acute alcohol-related hepatitis is an acute syndrome that frequently affects younger patients with unfavorable prognosis in severe cases that do not respond to medical therapy. In this setting, liver transplantation is feasible with excellent survival outcomes in selected patients. Return to alcohol use after LT is rare and there is a similar rate of patients with alcohol-related liver disease who underwent LT after 6 months of proven abstinence. The patient’s selection criteria are crucial to ensure better outcomes, and specialized psychologists and substance disorders specialists must be involved in a multi-disciplinary transplant team to optimize LT evaluation. Disparities among liver transplant centers are still present, leading to unequal access to liver transplantation. Future prospective studies are necessary to standardize the selection criteria for eLT and better predict the return to alcohol use after liver transplantation. Finally, further efforts are needed to overcome stigmas surrounding alcohol-related liver disease in order to standardize the allocation systems and ensure the same possibilities for all patients.

## Figures and Tables

**Table 1:** Emerging therapies in AH.

Therapy	Outcome	Reference/Trial
Anti-TNFα	Higher mortality rates due to increased risk of infection.	[52,53]
Pentoxifylline	No benefit on mortality over placebo, even when administered with steroid therapy.	[44]
IL-1 inhibitor (Anakinra)	No survival advantages were reported compared with steroid therapy.	[54]
N-acetylcysteine	In combination with a steroid, it improves survival.	[55,56]
Methadoxin	Improved survival at 3 and 6 months in combination with steroid.	[57]
Fecal microbiota transplantation (FMT)	Effective and safe in patients with severe AH, resulting in improved 1-year survival.	[58]

**Table 2:** Patients’ selection criteria in studies evaluating early liver transplantation in severe alcohol hepatitis.

Authors (Year)	Number of Patients	Study Design	Location	Patients’ Selection Criteria	Selection Rate	Survival Rate in eLT
Mathurin et al., 2011 [65]	28	Prospective Case–Control Study	France and Belgium. Multicentric	- Severe alcoholic hepatitis * as the first liver-decompensating event; - No co-morbidities (including psychiatric); - Good social support - Absolute consensus in a multidisciplinary setting	<2%	77 ± 8% at 6 months and 719% at 24 months
Im et al., 2016 [66]	9 (2/9 underwent liver-kidney LT for hemodialysis > 12 w)	Prospective Case–Control Study	United States. Monocentric	- Severe alcoholic hepatitis as the first liver-decompensating event * - Good social support - Signed agreement to lifelong alcohol abstinence - Excluded patients with concomitant chronic liver disease - No co-morbidities - Psychiatric disorders (previously/controlled or newly diagnosed depression or anxiety disorders) were not excluded - Recent infection and gastrointestinal bleeding not excluded	21%	89% at 6 months
Lee, B.P. 2017 [67]	17	Retrospective pilot study	United States. Monocentric	- Severe alcoholic hepatitis as the first liver-decompensating event * - No concomitant chronic liver disease - psychiatric disorders (previously/controlled or newly diagnosed depression or anxiety disorders) were not excluded - Recent infection and gastrointestinal bleeding not excluded	6.3%	100% at 6 months
Lee, B.P. 2018 [68]	147	Retrospective	United States. Multicentric (12 centers)	- Clinically diagnosed severe acute AH * - Excluded patients with concomitant chronic liver disease - Good social support - No co-morbidities - Recent infection and gastrointestinal bleeding not excluded - Only 2 centers excluded patients with previous psychiatric disease (even if well controlled)	35.9%	94% at 1 year and 84% at 3 years.
Lee B.P. 2022 [69]	153	Retrospective	United States. Multicentric (11 centers)	- Severe alcoholic hepatitis * - excluded patients with concomitant chronic liver diseases - No specific period of abstinence	Not reported	95%, 88% and 82%. At 1, 3 and 5 years
Louvet 2022 [70]	68	Prospective, non-randomized non-inferiority controlled trial	France and Belgium. Multicentric (2 centers)	- Severe alcoholic hepatitis * (biopsy proven in most cases) assigned to the early transplantation group vs. not eligible for the early transplantation group if: - High alcohol intake, clinical diagnosis of alcohol-related hepatitis, - Hospitalized for less than 1 month	68%	89% at 2 years
Germani 2022 [71]	16	Prospective	Italy-multicentric (4 centers)	- Severe alcoholic hepatitis * - Gastrointestinal bleeding not excluded - Psychiatric disorders (previously/controlled or newly diagnosed depression or anxiety disorders) were not excluded.	38.5%	100% at 2 years

* Severe alcoholic hepatitis defined as Maddrey score > 32; non-responding to medical therapy (Lille > 0.45) chronic liver diseases (e.g., hepatitis virus B or C infection and those with hepatocellular carcinoma).

**Table 3:** Salt score.

Salt Score	
>10 drinks per day at diagnosis	+4 points
>2 failed rehabilitation attempts	+4 points
Any episodes of alcohol-related legal issue	+2 points
History of illicit substance abuse (except for THC)	+1 Point

SALT of ≥5 → 25% PPV of Post LT alcohol use; SALT < 5 → 95% NPV of Post LT alcohol use.

## Data Availability

No new data were created or analyzed in this study. Data sharing is not applicable to this article.
